# Distinct patterns of health risk behaviours in adolescents with thalassaemia

**DOI:** 10.1080/07853890.2025.2521441

**Published:** 2025-06-19

**Authors:** Pattaporn Kaewkong, Nonglak Boonchooduang, Narueporn Likhitweerawong, Pimlak Charoenkwan, Orawan Louthrenoo

**Affiliations:** a Department of Pediatrics, Chiang Mai University, Chiang Mai, Thailand; b Thalassemia and Hematology Center, Chiang Mai University, Chiang Mai, Thailand

**Keywords:** Thalassaemia, adolescent health, risk-taking behaviour, chronic disease, mental health

## Abstract

**Background:**

Thalassaemia is a significant public health concern in Southeast Asia, yet little is known about health risk behaviours in adolescents with this condition. This study aimed to compare health risk behaviours between adolescents with thalassaemia and healthy controls, and to identify associated clinical and sociodemographic factors.

**Methods:**

This cross-sectional study included 60 adolescents with thalassaemia (aged 10–18 years) and 60 age- and sex-matched healthy controls in Thailand. Health risk behaviours were assessed using the validated Thai Youth Risk Behaviour Survey. Clinical data were extracted from medical records. Multivariable logistic regression was used to identify factors associated with risk behaviours.

**Results:**

Adolescents with thalassaemia reported higher rates of non-seatbelt use (25.0% vs. 1.7%, *p* < 0.001) and mental health problems (13.3% vs. 0.8%, *p* = 0.027) compared to controls. However, they exhibited lower rates of inadequate exercise (70.0% vs. 86.7%, *p* = 0.045) and excessive screen time (66.7% vs. 96.7%, *p* < 0.001). In the thalassaemia group, older age (aOR = 2.78, 95% CI: 1.09–7.09), female gender (aOR = 39.73, 95% CI: 0.89–1770.49) and lower pre-transfusion haemoglobin levels (aOR = 0.09, 95% CI: 0.01–0.64) were associated with increased odds of mental health problems. Higher pre-transfusion haemoglobin levels were associated with lower odds of violent behaviour (aOR = 0.44, 95% CI: 0.21–0.92).

**Conclusions:**

Adolescents with thalassaemia exhibit distinct patterns of health risk behaviours, influenced by age, gender and disease severity. These findings underscore the need for integrated physical and psychosocial care, highlighting the importance of tailored health education and interventions for this vulnerable group.

## Introduction

Thalassaemia, a group of inherited haemoglobinopathies characterized by impaired globin chain synthesis, represents a significant public health concern in Southeast Asian countries, including Thailand [1]. The global prevalence of thalassaemia carriers is estimated at 1.5%, with carrier rates as high as 25% in certain regions such as Southeast Asia [2]. In Thailand, approximately 1% of the population has a severe form of thalassaemia [3].

Advances in medical care have led to a substantial improvement in the life expectancy of individuals with thalassaemia, with many now surviving into adulthood [4,5]. However, the chronic nature of the disease and its treatment, which often involves regular blood transfusions and iron chelation therapy, can have a profound impact on various aspects of patients’ lives, including their physical, psychological and social well-being [6,7].

Adolescence, a critical period of development characterized by rapid physical, emotional and social changes [8], presents unique challenges for adolescents with thalassaemia. These challenges are augmented by the demands of managing a chronic illness [9]. Previous studies have highlighted the increased risk of mental health problems, such as depression and anxiety, among adolescents with thalassaemia compared to their healthy peers [10,11]. However, less attention has been given to the prevalence and correlates of health risk behaviours in this population.

Health risk behaviours, such as substance use, risky sexual activity and violence, are major concerns for adolescents globally. These behaviours can have serious long-term consequences, including addiction, injuries, legal issues, chronic diseases and lasting psychological trauma. Additionally, these risk behaviours often continue into adulthood. Behaviours such as tobacco and alcohol use, physical inactivity and poor diet, which emerge during adolescence, significantly increase the risk for major non-communicable diseases (NCDs) such as cardiovascular disease and type 2 diabetes later in life [12]. Studies have suggested that adolescents with chronic illnesses may be at increased risk of engaging in health risk behaviours, possibly due to factors such as stress, social isolation, or a desire for normalization [13,14]. However, the majority of these studies have focused on chronic illnesses in general, rather than specifically on thalassaemia.

To date, there is limited research on the prevalence and correlates of health risk behaviours among adolescents with thalassaemia, particularly in Southeast Asian countries where the disease is most prevalent. A better understanding of these behaviours and their associated factors is crucial for developing targeted interventions and support services for this vulnerable population.

This study aims to address these gaps in the literature by: (1) comparing the prevalence of health risk behaviours between adolescents with thalassaemia and healthy controls in Thailand; and (2) identifying clinical and sociodemographic factors associated with these behaviours in the thalassaemia group. We hypothesize that adolescents with thalassaemia will have higher rates of certain health risk behaviours compared to controls and that these behaviours will be associated with greater disease severity.

The novelty of this study lies in its comprehensive assessment of health risk behaviours in adolescents with thalassaemia, a population that has been understudied in this context, especially in Southeast Asia. The findings will provide valuable insights into the challenges faced by adolescents with thalassaemia and inform the development of comprehensive care strategies that address both their physical and psychosocial needs.

## Materials and methods

### Study design and participants

This cross-sectional study was conducted at the Outpatient Clinic of Pediatric Hematology, Thalassemia and Hematology Center, at Chiang Mai University Hospital in Thailand between February and November 2024. The study included 60 adolescents with a confirmed diagnosis of thalassaemia who were receiving regular follow-up care at the clinic. The age range of 10–18 years was selected to capture the entire adolescent period, including early, middle and late adolescence, which are characterized by distinct developmental challenges. A control group of 60 healthy adolescents, matched for age and sex, was recruited from the general paediatric clinic during the same period using a convenience sampling method.

Inclusion criteria for both groups were: (1) age between 10 and 18 years; (2) ability to read and write in Thai; and (3) availability of a parent or legal guardian to provide informed consent. Exclusion criteria for both groups included: (1) history of chronic illness other than thalassaemia; (2) presence of cognitive impairment; and (3) diagnosed psychiatric disorder.

The sample size was calculated based on a previous study that found a prevalence of depression of 38.5% among adolescents with chronic illness compared to 15.9% in healthy controls [15]. With a power of 80% and a two-sided alpha of 0.05, a minimum of 60 participants per group was required to detect this difference.

### Assessment of health risk behaviours

All participants completed a set of electronic questionnaires administered through a secure online platform. The use of electronic questionnaires minimized data entry errors and ensured completeness of responses. To ensure privacy, participants accessed the questionnaires through a unique link provided to them and completed the assessments on a private electronic device during their clinic visit. The Thai Youth Risk Behavior Survey (YRBS), adapted from the US Centres for Disease Control and Prevention, assesses health risk behaviours in the past 12 months across six domains: unintentional injuries, violence acts, substance use, mental health problems, sexual behaviours and unhealthy behaviours [16]. The Thai version of the YRBS has been previously validated in adolescent populations, demonstrating good reliability and validity [17].

Mental health problems were identified based on responses to YRBS items assessing emotional distress, including persistent sadness or hopelessness, suicidal ideation and suicide attempts within the past 12 months. Formal clinical psychological evaluations were not conducted; all data on mental health problems reflect self-reported experiences.

Adequate physical activity was defined as engaging in at least 60 min of moderate-to-vigorous physical activity on five or more days per week, in accordance with World Health Organization (WHO) guidelines [18]. Excessive screen time was defined as more than 2 hours per day of recreational (non-educational) screen use, based on recommendations from the American Academy of Pediatrics [19].

For participants with thalassaemia, clinical data were extracted from their medical records, including type of thalassaemia, age at diagnosis, transfusion history and iron chelation therapy. Pretransfusion haemoglobin and serum ferritin levels within the past 6 months were also recorded.

### Statistical analysis

Data were analyzed using SPSS version 26.0 (IBM Corp., Armonk, NY, USA). Continuous variables were summarized as means and standard deviations for normally distributed data or medians and interquartile ranges for non-normally distributed data. Categorical variables were presented as frequencies and percentages. Differences between the thalassaemia and control groups were examined using independent t-tests for normally distributed continuous variables, Mann-Whitney U tests for non-normally distributed continuous variables, and chi-square tests for categorical variables.

Within the thalassaemia group, associations of clinical and sociodemographic factors with YRBS risk behaviour domains were explored using multivariable logistic regression models. Adjusted odds ratios (aOR) with 95% confidence intervals (CIs) were reported. The Hosmer-Lemeshow test was used to assess model fit, and variance inflation factors were calculated to check for multicollinearity. Statistical significance was set at *p* < 0.05 for all analyses.

### Ethical considerations

This study was conducted in accordance with the Declaration of Helsinki, and the protocol was approved by the Institutional Review Board of the Faculty of Medicine, Chiang Mai University (IRB No. 061/2024). Written informed consent was obtained from all participants’ parents or legal guardians, and written assent was obtained from the participants themselves. Confidentiality of the participants’ information was maintained throughout the study.

## Results

### Participant characteristics

A total of 120 adolescents (60 with thalassaemia and 60 healthy controls) participated in the study. The mean age was 13.4 years (SD = 2.6) in the thalassaemia group and 13.4 years (SD = 1.8) in the control group (*p* = 0.94). Both groups had an equal proportion of males (58.3%). There were significant differences between the groups in terms of weight, height and mean BMI, with the thalassaemia group showing lower values in all three parameters. However, there were no significant differences in parental marital status between the groups (Table 1).

**Table 1.** Demographic characteristics of participants.

	Thalassaemia group (*n* = 60)		Healthy group (*n* = 60)		*p*-value
Male gender, *n* (%)	35	(58.3)	35	(58.3)	>0.999
Age(y), mean (SD)	13.4	(2.6)	13.42	(1.8)	0.94
Weight (kg), mean (SD)	39.9	(11.0)	50.2	(9.1)	<0.001
Height (cm), mean (SD)	148.2	(13.3)	156.8	(10.8)	<0.001
BMI (kg/m^2^), mean (SD)	17.8	(2.9)	20.4	(2.7)	<0.001
Parental marital status, *n* (%)					
Couple	47	(78.3)	4	(80.0)	>0.999
Divorce/single parent	13	(21.7)	12	(20.0)	
Maternal education level, *n* (%)					0.001
Middle school or lower	14	(23.3)	2	(3.3)	
High school or Vocation certificate	18	(30.0)	13	(21.7)	
Bachelor’s degree or higher	28	(46.7)	45	(75.0)	
Paternal education level, *n* (%)					0.008
Middle school or lower	14	(23.3)	3	(5.0)	
High school or Vocation certificate	17	(31.7)	17	(28.3)	
Bachelor’s degree or higher	27	(45.0)	40	(66.7)	

BMI: body mass index.

Among the adolescents with thalassaemia, the majority had Haemoglobin E/β-thalassaemia (55.0%), followed by Haemoglobin H disease (23.3%), β-thalassaemia major (11.7%) and AE Bart’s disease (10.0%). The mean age at diagnosis was 2.17 years (SD = 3.17). Most participants were transfusion-dependent (70.0%) and receiving iron chelation therapy (68.4%), with deferasirox being the most common chelator (46.7%). The mean pre-transfusion haemoglobin level was 8.95 g/dL (SD = 1.29), and the mean serum ferritin level was 1,776 µg/L (SD = 1,431). Complications were observed in 40% of cases, including transaminitis, symptomatic gallstones, short stature, hypopituitarism, hypersplenism, vitamin D deficiency and autoimmune haemolytic anaemia (AIHA) (Table 2).

**Table 2.** Clinical characteristics of adolescents with thalassaemia (*n* = 60).

Clinical characteristics		
Type of thalassaemia, *n* (%)		
β-thalassaemia major	7	(11.7)
Haemoglobin E/β-thalassaemia	33	(55.0)
Haemoglobin H disease	14	(23.3)
AE Bart’s disease	6	(10.0)
Age at diagnosis (y), mean (SD)	2.17	(3.17)
Transfusion dependency, *n* (%)		
Non-transfusion dependent thalassaemia (NTDT)	18	(30.0)
Transfusion dependent thalassaemia (TDT)	42	(70.0)
Pre-transfusion haemoglobin level (g/dL), mean (SD)	9	(1.3)
Serum ferritin level (µg/L), mean (SD)	1,776	(1,431)
Iron chelator, *n* (%)		
None	19	(31.7)
Deferiprone	7	(11.7)
Deferasirox	28	(46.7)
>1 iron chelation	6	(10.0)
Age at initiation of regular transfusion (y), mean (SD)	3.24	(3.80)
Age at first iron chelation (y), mean (SD)	5.22	(4.65)
Complication, *n* (%)	24	(40.0)

### Prevalence of health risk behaviours

**Figure 1.**
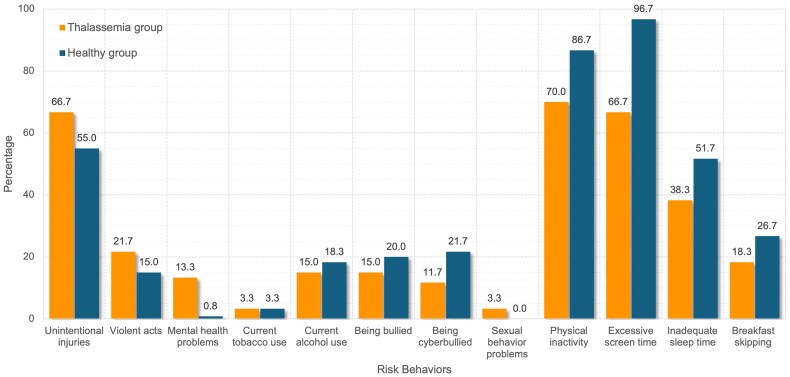
Prevalence of health risk behaviours in adolescents with thalassaemia and healthy controls.

**Figure 2.**
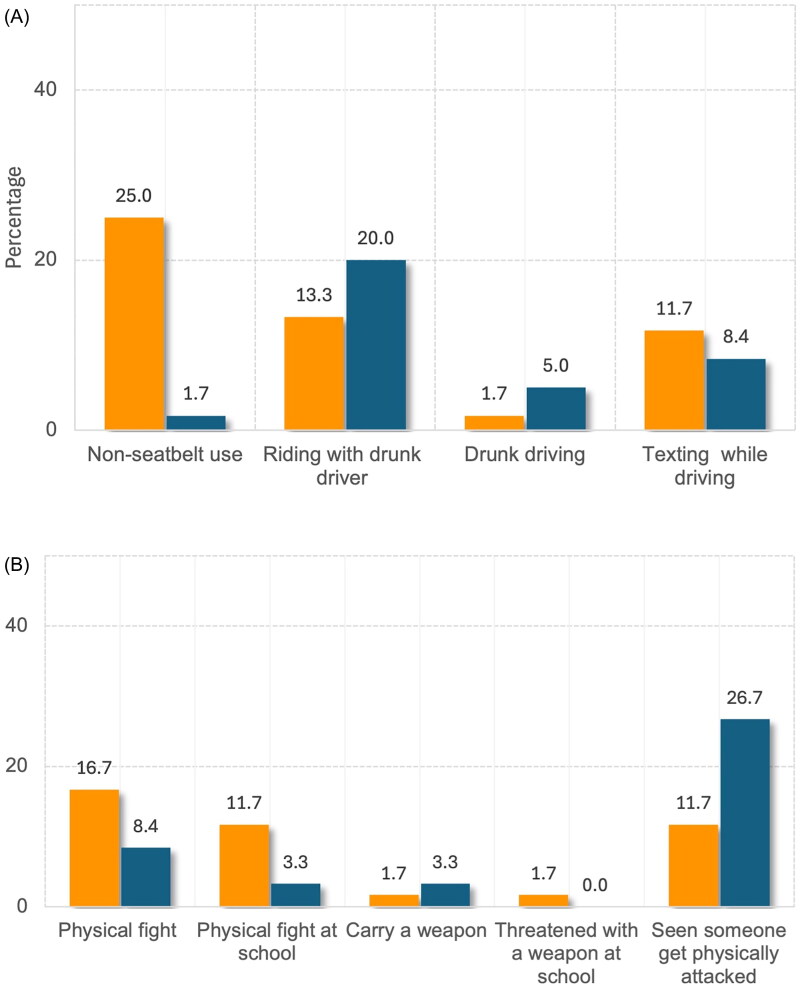
Prevalence of (a) unintentional injuries, (b) violent acts in adolescents with thalassaemia and healthy controls.

Adolescents with thalassaemia were significantly more likely to report non-seatbelt use compared to healthy controls (25.0% vs. 1.7%, *p* < 0.001) (Figure 2a). They also had higher rates of self-reported mental health problems, including persistent sadness, suicidal ideation, or suicide attempts (13.3% vs. 0.8%, *p* = 0.027). The control group showed higher rates of inadequate exercise (86.7% vs 70.0%, *p* = 0.045) and excessive screen time (96.7% vs. 66.7%, *p* < 0.001). There were no significant differences between the groups in other risk behaviours, including substance use, violence-related behaviours, suicidality, sexual activity and unhealthy dietary habits (Figure 1). While there was no statistically significant difference, the rate of violent acts in thalassaemia adolescents was higher than in healthy peers, especially regarding physical fights (Figure 2b).

### Factors associated with health risk behaviours in the thalassaemia group

Multivariable logistic regression analysis revealed several significant predictors of mental health problems in adolescents with thalassaemia. Older age (aOR = 2.78, 95% CI: 1.09–7.09, *p* = 0.032), higher paternal education (aOR = 36.63, 95% CI: 1.94–692.7, *p* = 0.016) and being female (aOR = 39.73, 95% CI: 0.89–1770.49, *p* = 0.057) were associated with increased odds of mental health problems. While higher pre-transfusion haemoglobin levels (aOR = 0.09, 95% CI: 0.01–0.64, *p* = 0.016) and higher BMI (aOR = 0.45, 95% CI: 0.20–0.99, *p* = 0.046) were associated with decreased odds.

**Figure 3.**
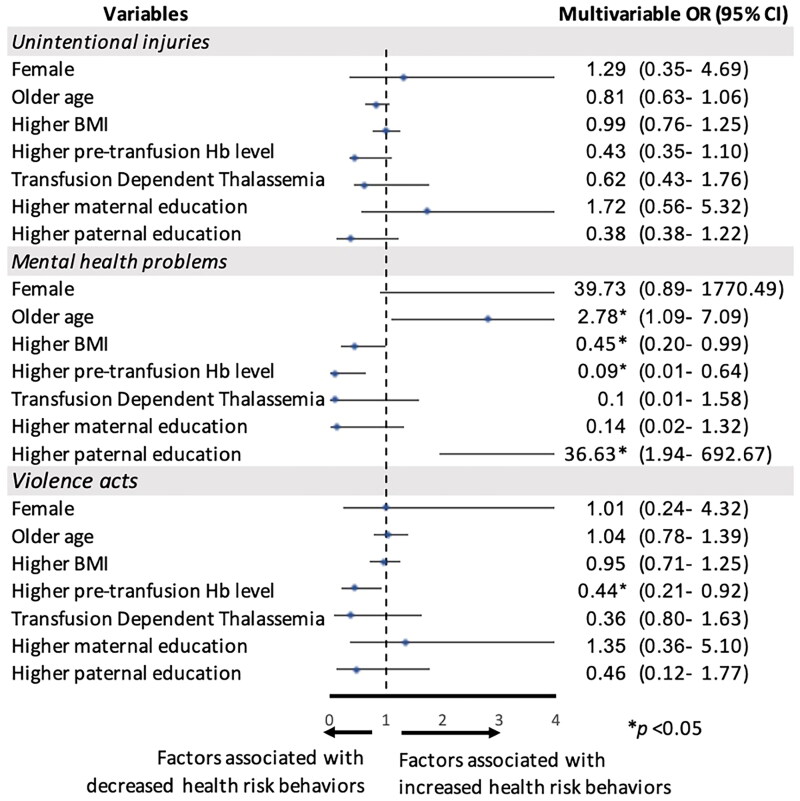
Factors associated with unintentional injuries, mental health problems and violent acts in adolescents with thalassaemia. The Forest plot displays adjusted odds ratios (aORs) and their 95% confidence intervals (CIs) for various factors associated with health risk behaviours. The vertical line at 1 represents no association. Points to the right of this line (aOR > 1) indicate increased odds of the behaviour, while points to the left (aOR < 1) indicate decreased odds. The horizontal lines represent 95% CIs; if a line crosses the vertical line at 1, the association is not statistically significant at the *p* < 0.05 level. Wider CIs suggest less precise estimates. Statistically significant associations are marked with asterisks. BMI: body mass index, Hb: haemoglobin

Higher pre-transfusion haemoglobin levels (aOR = 0.44, 95% CI: 0.21–0.92, *p* = 0.030) were significantly associated with lower odds of engaging in violent acts among adolescents with thalassaemia. The logistic regression model did not identify any significant predictors of unintentional injuries among adolescents with thalassaemia (Figure 3).

### Model diagnostics

The Hosmer-Lemeshow test was non-significant for all models (mental health problems χ^2^ = 6.234, *p* = 0.621; violent acts χ^2^ = 10.496, *p* = 0.232; unintentional injuries χ^2^ = 5.972, *p* = 0.650), supporting the adequacy of the model fit. Collinearity diagnostics for the predictors in the regression models showed Tolerance values greater than 0.1 and Variance Inflation Factor (VIF) values below 5 for all predictors, indicating that multicollinearity was not a concern.

## Discussion

This study reveals distinct patterns of health risk behaviours among adolescents with thalassaemia, underscoring the unique interplay between chronic illness and adolescent development. Adolescents with thalassaemia in our study exhibited a higher prevalence of mental health problems compared to healthy controls. This finding aligns with previous research on chronic illnesses in adolescence [20,21]. Borgna-Pignatti et al. reported that as survival rates improve for individuals with thalassaemia, psychosocial complications become increasingly important [22]. We found a significant association between lower pre-transfusion haemoglobin levels and mental health problems, suggesting a potential biological mechanism linking disease severity to psychological well-being. This relationship is supported by prior studies linking lower haemoglobin levels to increased mental health problems in young adults with thalassaemia major [10]. These results underscore the importance of optimal disease management for both physical and mental health outcomes.

Interestingly, while mental health concerns were elevated, adolescents with thalassaemia generally reported lower engagement in externalized health risk behaviours, such as substance use, violence and sexual activity, compared to controls. This apparent inverse relationship may reflect protective influences—such as frequent medical supervision, close parental involvement and illness-related coping strategies—that encourage behavioural caution despite internal distress. Understanding these protective mechanisms can inform supportive interventions that enhance resilience while addressing emotional vulnerabilities.

For example, our findings on physical activity showed that adolescents with thalassaemia were more active than their healthy peers—a pattern contrary to studies in other chronic illnesses such as congenital heart disease or juvenile idiopathic arthritis [23,24]. Our finding may reflect increased health awareness or the benefits of regular medical follow-up, though it remains below the recommended levels for adolescents [25]. This suggests that tailored health programs could further improve exercise participation among adolescents with thalassaemia.

We also observed an association between higher BMI and lower odds of mental health problems in our thalassaemia group, diverging from findings in other chronic illnesses where higher BMI often correlates with poorer mental health outcomes [26]. This may highlight the importance of adequate nutrition and positive body image, particularly in a condition where growth delays and body image issues are common concerns.

Furthermore, screen time usage in adolescents with thalassaemia was significantly lower than in their healthy counterparts (66.7% vs. 96.7%, *p* < 0.001). This contrasts with the general trend of high screen time usage among adolescents, particularly those with chronic illnesses [19]. Several factors may contribute to this observation. The time-consuming nature of thalassaemia management, including regular hospital visits, blood transfusions and daily medication regimens, may limit the available time for screen-based activities. Additionally, parents of children with thalassaemia may impose stricter limits on screen time as part of an overall health-conscious approach to their child’s well-being. However, it’s important to note that while lower than their healthy peers, the rate of excessive screen time in the thalassaemia group remains high at 66.7%. This suggests that screen time management should still be addressed in thalassaemia care. The impact of screen time on sleep patterns, physical activity and overall quality of life in this population warrants further investigation. Moreover, the quality of screen time, rather than just quantity, should be considered. Digital platforms can offer valuable resources for disease education, peer support and health management tools for adolescents with chronic conditions [19,27]. Future research should explore the patterns of digital media use in this population, distinguishing between educational, social and entertainment purposes, to inform guidelines for healthy digital habits in adolescents with thalassaemia.

An unexpected and concerning finding was the elevated rate of non-seatbelt use among adolescents with thalassaemia compared to healthy controls, contrasting with trends seen in other chronic illnesses where safety-related risk behaviours are often similar or lower relative to healthy peers [28,29]. This behaviour may reflect a sense of fatalism as a coping mechanism for their chronic condition [13]. Additionally, these adolescents may rebel against the constant health-related restrictions imposed by their condition, manifesting as risk-taking behaviours in areas they perceive as less directly related to their illness [30]. Furthermore, the intense focus on disease-specific care in thalassaemia management might inadvertently lead to a relative neglect of general safety education [31]. This highlights the need for a more comprehensive approach to health education in chronic illness care, addressing not only disease management but also general health and safety practices.

Interestingly, we found no significant differences between groups in substance use, violence-related behaviours, or sexual activity, which is reassuring. However, the overall rates of some of these behaviours were low in both groups, which may have limited our ability to detect differences. This underscores the importance of continued monitoring and preventive interventions for all adolescents, regardless of their health status.

Our analysis revealed several significant associations between sociodemographic factors and mental health problems in adolescents with thalassaemia. Older age, female gender and lower pre-transfusion haemoglobin levels were associated with increased odds of mental health problems. These findings align with broader adolescent health literature and previous studies on thalassaemia [22,32,33].

The association between higher paternal education and increased mental health problems in our thalassaemia group warrants careful interpretation. Several confounding factors may influence this relationship, including socioeconomic status, parental expectations, family dynamics and access to mental health awareness. Higher educational attainment may be associated with greater awareness and reporting of mental health concerns rather than actual increased prevalence [11]. Alternatively, it could be related to higher academic expectations placed on children managing a chronic illness, potentially contributing to increased stress and anxiety. This finding, however, contrasts with studies from Western contexts, where higher parental education often correlates with better mental health outcomes [9]. The discrepancy might be attributed to cultural differences in parenting styles, educational expectations, or the perceived impact of chronic illness on future prospects. Further exploration in cultural contexts would be valuable.

Our findings have direct implications for clinical practice in thalassaemia care. We recommend implementing routine mental health screening using brief validated tools during regular thalassaemia follow-up visits, particularly targeting older adolescents and those with lower pre-transfusion haemoglobin levels. Safety education, with special emphasis on seatbelt use, should be integrated into standard health counselling protocols. Multidisciplinary teams including mental health professionals should be available for referral when screening identifies concerns. Additionally, peer support programs could be beneficial for adolescents struggling with illness-related stressors. Finally, transition programs for older adolescents should address both disease management skills and broader health behaviours to support long-term well-being as patients move to adult care.

The study has several strengths, including the use of validated measures, a well-matched control group, and the examination of a range of health risk behaviours. However, there are also some limitations to consider. The cross-sectional design precludes conclusions about causality, and the reliance on self-report measures may have introduced bias. While the use of electronic questionnaires minimizes some biases, self-reported data remains susceptible to social desirability bias. Adolescents might underreport behaviours they perceive as negative or overreport positive behaviours. The sample size, while adequate for our primary analyses, may have been insufficient to detect smaller differences in less common behaviours. Additionally, the single-centre nature of our study may limit its generalizability.

Future research should include longitudinal studies to track how risk behaviours evolve over time in adolescents with thalassaemia, ideally extending the age range up to 25 years to better understand the transition to adulthood and account for cultural aspects around independent living and sexual behaviours in the Thai population.

Future studies could incorporate objective measures, such as activity trackers or clinical assessments, to validate self-reported data. Interventional studies testing targeted programs for safety education, mental health support and physical activity promotion in this population would be valuable. Qualitative research exploring the lived experiences of adolescents with thalassaemia could provide deeper insights into the mechanisms underlying the observed patterns of risk behaviours.

## Conclusion

This study highlights the unique pattern of health risk behaviours in adolescents with thalassaemia, emphasizing the need for a holistic approach to care that addresses both physical and psychosocial needs. By integrating mental health support, safety education and lifestyle counselling into routine thalassaemia care, clinicians may significantly improve the overall health outcomes and quality of life for this vulnerable population. A multi-disciplinary approach involving paediatricians, psychologists, nutritionists and educators is essential for addressing the comprehensive needs of adolescents with thalassaemia as they transition to adult care.

## Data Availability

The data that support the findings of this study are available from the corresponding author upon reasonable request.
